# Longitudinal polarization diversity optical coherence tomography before and after photodynamic therapy in chronic central serous chorioretinopathy: a prospective clinical study

**DOI:** 10.1186/s40942-026-00857-8

**Published:** 2026-05-11

**Authors:** Parsa Khatami, Mohammad Shahidul Islam, Thomas J. van Rijssen, Matheus Rizzo, Iago Rocha Bastos, Giulianna Mendes Recchia, Ali Tavakoli, Farhad Tafreshi, Myeong Jin Ju, Eduardo Vitor Navajas

**Affiliations:** 1 Faculty of Medicine, University of British Columbia, Vancouver, British Columbia, V6T 1Z3 BC Canada; 2 School of Biomedical Engineering, Faculty of Medicine and Applied Science, University of British Columbia, Vancouver, BC V6T 1Z3 Canada; 3 Department of Ophthalmology, Leiden University Medical Center, Leiden, Netherlands; 4 Department of Ophthalmology and Visual Sciences, University of British Columbia, 2550 Willow St, Eye Care Centre, Section D, Vancouver, BC V6T 1Z3 Canada; 5 Faculty of Science, University of British Columbia, Vancouver, British Columbia, BC V6T 1Z4 Canada

**Keywords:** Polarization-diversity optical coherence tomography, Chronic central serous chorioretinopathy, Photodynamic therapy, Retinal pigment epithelium, Melanin, Choriocapillaris, DOPU

## Abstract

**Background:**

Central serous chorioretinopathy (CSC) is the fourth most common retinopathy globally. Chronic central serous chorioretinopathy (cCSC) is a subtype of CSC characterized by persistent or recurrent subretinal fluid (SRF) for more than 3 months. CSC’s pathophysiology is described by choriocapillaries (CC) hyperpermeability and vascular congestion. Photodynamic therapy (PDT) is the current treatment of choice for cCSC. PDT causes vascular remodelling by inducing reactive oxygen species (ROS). Melanin in the retinal pigment epithelium (RPE) is a major target of ROS and can be observed non-invasively using polarization-diversity OCT (PD-OCT). Fourier-domain mode-locked (FDML) OCT is an imaging modality that can be used for high-resolution imaging of choriocapillaries.Our study aimed to evaluate melanin-related depolarization and CC changes longitudinally before and after PDT in patients with cCSC.

**Methods:**

This prospective clinical pilot study was conducted in accordance with the Declaration of Helsinki and approved by the institutional review board (REB H15-02914). 5 eyes with cCSC were imaged at baseline and within one hour, one week, and one month after half-dose PDT. Eligible patients had cCSC with active disease on multimodal imaging and no other major ocular comorbidities. Imaging included custom-built swept-source OCT angiography (OCTA) and polarization-diversity OCT (PD-OCT) and commercial SSOCT. PD-OCT depolarization was qualitatively assessed using degree of polarization uniformity (DOPU). PD-OCT volumes were manually segmented into: (1) the RPE layer (Inner RPE to outer Bruch’s membrane), the full choroidal slab (Outer RPE to Choroidoscleral interface).

**Results:**

PD-OCT revealed a decrease in DOPU after PDT across all patients. Manual segmentation of PD-OCT images showed that this effect was most prominent within the RPE-Bruch’s Complex. The full choroidal slab demonstrated less depolarization post-PDT. FDML OCTA demonstrated transient flow alterations in the choriocapillaris within the same 4,000-µm PDT treatment zone. Immediately after PDT, FDML revealed irregular flow deficits and heterogeneous darkening across the choriocapillaris.

**Conclusion:**

DOPU provides a novel, non-invasive marker for assessing RPE melanin integrity and may serve as a complementary tool for DOPU related changed following PDT in CSC. Our findings suggest a novel mechanism and transient effect of PDT action on RPE melanin.

**Supplementary Information:**

The online version contains supplementary material available at 10.1186/s40942-026-00857-8.

## Background

Central serous chorioretinopathy (CSC) is the fourth most common retinopathy worldwide and it is characterized by serous detachment of the neural retina and/or retinal pigment epithelium (RPE) [1]. It commonly affects middle-aged adults between 30 and 50 years old. The main risk factors for CSC include male sex (odds ratio (OR) = 5.63) [2], endogenous glucocorticosteroids production, and exogenous corticosteroid intake such as oral, topical, intravitreal, intravenous, or nasal [3]. Two types of CSC are differentiated: acute and chronic (cCSC) [4]. In contrast to acute CSC, cCSC is not self-limiting and subretinal fluid (SRF) persists for more than 3 months [1]. cCSC leads to permanent structural alterations including thinning and reduced vessel density of the choriocapillaris, RPE hyperplasia and/or atrophy and photoreceptor layer damage.[5].

The precise pathogenesis of CSC is not known [6]. The choroid has been postulated as the primary site of pathology as evidenced by the presence of choroidal vascular hyperpermeability, venous dilation, delayed choroidal filling on indocyanine green angiography (ICGA). There is increased choroidal thickness, dilated Haller’s layer vessels on enhanced depth imaging spectral domain OCT (EDI-OCT), and swept source OCT (SS-OCT) [7]. The accumulation of fluid from increased CC leakage results in increased hydrostatic pressure in the choroidal compartment causing microrips in the overlying RPE and the formation of serous RD.[4].

The hyperpermeability of the choriocapillaris forms the rationale for the use of photodynamic therapy (PDT) in the treatment of cCSC [8]. PDT induces reduction of vascular hyperpermeability at the level of the choriocapillaris leading to resolution of SRF [8, 9]. The use of half dose (3 mg/m^2^) or half fluence (25J/cm^2^) have reduced the risks of overlying RPE damage with PDT but RPE atrophy after treatment is still possible [10, 11]. The laser used in PDT excites the photosynthesizing agent verteporfin and forms free radicals, including singlet Oxygen (^1^ O₂) [12, 13]. Singlet Oxygen remodels the endothelial cells and facilitates SRF reabsorption [13]. Interestingly,^1^ O₂-mediated oxidation is particularly strong at double bonds in the tyrosine-derived rings of melanin [14]. Therefore, it is possible that the PDT effect on the melanin in the sick RPE of cCSC patients is a predisposing factor for further damage leading to RPE atrophy. In addition, PDT also causes temporary reduction of CC flow which may also be a contributing factor through ischemia of the RPE.[15].

Polarization-sensitive OCT (PS-OCT) is an imaging modality that visualizes tissue based on its polarization properties [16]. Polarization-diversity OCT (PD-OCT) is a subtype of PS-OCT that combines polarization measurements with scattering information from conventional OCT [17]. PD-OCT uses the degree of polarization uniformity (DOPU) as a metric to quantify tissue polarization [17]. DOPU can be used to identify subtle changes in the RPE, such as microrips, aggregations, and skip lesions, which are often missed by conventional OCT [17]. In particular, melanin granules in the RPE do not preserve polarization but instead induce depolarization, increasing entropy and thereby lowering the measured DOPU value. The major source of changes in polarized light occurs at the level of melanin in the RPE.[18].

OCT angiography has been used to image and perform quantitative assessment of the choriocapillaris [19]. In cCSC patients, frame averaged OCTA of choriocapillaris revealed reduced number of capillaries with longer and wider vessels.[20].

The goal of this study was to utilize PD-OCT and OCTA to evaluate the effects of PDT in the RPE melanin and choriocapillaris of patients with cCSC.

## Method

### Patient selection and study design

This pilot prospective clinical trial was approved by the Institutional Review Board of the University of British Columbia (UBC), Vancouver, British Columbia, Canada (H15-02914) and adhered to the tenets of the Declaration of Helsinki. Procedures performed involving human participants were in accordance with the ethical standards of the institutional and/or national research committee and with the Helsinki Declaration and its later amendments. Written and verbal informed consents were obtained from all study participants.

A total of 12 patients with cCSC were imaged between May 2025 and September 2025; of these, five patients met all inclusion criteria and were included in the analysis. Participants were recruited from the Department of Ophthalmology and Visual Sciences, UBC and provided written informed consent prior to enrollment. Eligible patients with diagnosed cCSC were imaged in conjunction with their scheduled PDT appointment. Exclusion criteria included: (1) significant media opacities that compromised image quality; (2) active intraocular inflammation; (3) retinal or choroidal disease other than cCSC (e.g., age-related macular degeneration, drusen, retinal vascular occlusions, or macular structural abnormalities); (4) prior vitrectomy; (5) history of glaucoma, prior glaucoma surgery, or current use of intraocular pressure–lowering medications; and (6) any intraocular procedure within 3 months of baseline imaging (including cataract surgery, Nd:YAG capsulotomy, panretinal photocoagulation, or intravitreal injection of anti-VEGF agents or corticosteroids). Prior to imaging, pupils were dilated using 2.5% phenylephrine hydrochloride and 1% tropicamide.

At baseline, each participant underwent a standardized multimodal OCT imaging protocol. First, five consecutive OCT angiography (OCTA) volumes were acquired using the PLEX Elite 9000 OCTA system (Carl Zeiss Meditec, Dublin, CA) with 3 × 3 mm^2^ and 6 × 6 mm^2^ fields of view (FOVs). Next, polarization-diversity OCT (PD-OCT) imaging was performed, during which five volumetric OCT datasets were acquired over an 8 × 8 mm^2^ FOV with a sampling density of 600 (slow axis) × 600 (fast axis). Finally, participants were imaged using a custom-built 1.6 MHz Fourier-domain mode-locked (FDML) OCT system which became available during the study, acquiring five OCTA volumes over a 750 × 750 µm^2^ FOV with an effective sampling density of 300 (fast axis) × 300 (slow axis), following 4-buffer averaging and eight repeated B-scans (BM) per location. This multimodal imaging protocol was performed at baseline and repeated at three post-treatment time points: (i) approximately 1 hour after PDT, (ii) 1 week after PDT (structural OCT and OCTA only), and (iii) 1 month after treatment (full multimodal OCT imaging).

### Treatment protocol

All participants underwent half-dose PDT with verteporfin using a standardized protocol. Briefly, Verteporfin was administered intravenously at a dose of 3 mg/m^2^ body surface area over 10 minutes. Five minutes after completion of the infusion, a 689-nm diode laser was applied to areas of leakage identified on fluorescein angiography (FA), which was performed within 2 weeks of treatment. Laser parameters were set to deliver a total light dose of 50 J/cm^2^ at an irradiance of 600 mW/cm^2^ for 83 seconds. The laser spot size was defined as the greatest linear dimension of the leakage area (s) on FA plus an additional 1 mm margin, while avoiding the optic nerve head and the region within 500 µm of the optic disc margin. All procedures were performed under pharmacologic mydriasis using 0.5% tropicamide by an experienced retinal specialist, and patients subsequently underwent routine follow-up examinations with OCT imaging at predefined visits to assess anatomical and functional treatment response.

### Multimodal OCT imaging and quantitative assessment

#### Polarization-diversity (PD) OCT

A custom-built polarization-diversity OCT (PD-OCT) system was utilized for imaging. The system employed a vertical-cavity surface-emitting laser (VCSEL; SVM10F-0210, Thorlabs Inc., Newton, NJ, USA) centered at 1060 nm with a 100 nm sweep bandwidth and a 400 kHz A-scan rate, providing an axial resolution of 7.06 µm. Full system details have been described previously in [17]. For each eye, five consecutive volumetric datasets were acquired over an 8 × 8 mm^2^ field of view (FOV) with a sampling density of 600 (fast axis) × 600 (slow axis). Volumes were manually segmented at the outer border of the retinal pigment epithelium (RPE) by three independent graders (IB, GR, PK) using ITK-SNAP (v4.4.0; UNC and PICSL, University of Pennsylvania, USA) [21]. The resulting segmentations were exported and projected into en-face maps using a custom layer-specific visualization script, generating depth-resolved en-face images of the RPE and choroidal slabs.In addition to conventional structural contrast, the PD‑OCT system computed the degree of polarization uniformity (DOPU), a functional polarization metric sensitive to tissue depolarization properties. DOPU quantifies the uniformity of the polarization state of backscattered light over local neighborhoods, serving as a surrogate for depolarization caused by multiple scattering and tissue composition differences. Lower DOPU values indicate greater depolarization, which has been shown to provide contrast in melanin‑rich layers such as the RPE and choroid. DOPU images were generated from the PD signals by processing orthogonal polarization components and computing local polarization variance, followed by appropriate noise correction and *en‑face* projection to visualize spatial variations in depolarization contrast across the retina and choroid.

#### Commercial OCTA

OCTA imaging was performed using the commercial PLEX Elite 9000 system (Carl Zeiss Meditec, Dublin, CA) with 3 × 3 mm^2^ and 6 × 6 mm^2^ FOVs. Volumes were acquired with a 500 × 500 sampling grid at an A-scan rate of 200 kHz. The imaging depth range was 3 mm, with axial and transverse resolutions of 6.3 µm and 20 µm, respectively. The built-in fixation-tracking system was used to mitigate motion artifacts from microsaccades and blinking during acquisition. Image quality was assessed using the manufacturer-reported signal strength index, and only scans with a signal strength of ≥8/10 were included in the analysis. Quantitative structural parameters were evaluated using standardized definitions. All measurements were performed manually by a single masked grader (IB) at the foveal center using the calliper measurement tool within the Plex Elite 9000 (Carl Zeiss Meditec, Dublin, CA) review software, following previously described measurement protocols [22–25]. Central retinal thickness (CRT) was measured in non-resolving CSC as the distance between the inner limiting membrane (ILM) and the inner border of the ellipsoid zone (EZ) [22]. Second band thickness (SBT) was defined as the distance from the inner EZ border to the outer edge of subretinal debris when SRF was present, or to the outer EZ border when debris had resolved after PDT [22]. Subfoveal choroidal thickness (SFCT) was measured from the inner surface of Bruch’s membrane to the innermost choroid–sclera interface [23]. Subretinal fluid height was assessed as the distance between the outer photoreceptor border and the inner RPE layer border [22, 24]. CC and Choroidal hyperreflectivity was independently graded as normal or abnormal pre and post PDT on structural OCT images by two ophthalmology fellows.[25].

#### FDML OCT

A *posteriori*, A custom‑built 1.6‑MHz Fourier‑domain mode‑locked (FDML) OCT system (NG‑FDML‑1060‑4B‑FA, OptoResGmbH, Germany) was incorporated into the study to complement commercial OCTA and PD‑OCT by providing high‑speed, high‑density volumetric angiography capable of detailed visualization of the microvascular network, especially the choriocapillaris. The system operated at a central wavelength of 1060 nm with a 75-nm full-width at half maximum (FWHM) sweep bandwidth, providing a theoretical axial resolution of ~6.18 µm in air. A zoom fiber collimator (ZC618APC-B, Thorlabs Inc., Newton, NJ, USA) was used to adjust the beam diameter (1/e^2^) from 1.12 to 3.47 mm, corresponding to transverse retinal resolutions ranging from 19.28 µm to 6.22 µm, respectively. Incident power at the cornea was maintained at ~3.0 mW, within ANSI safety limits at 1060 nm [26]. To maximize raster scanning duty cycle while maintaining a consistent interscan interval, which is critical for the microvascular details, a step-bidirectional scanning strategy was implemented.[27].

OCTA volumes were acquired over a 750 × 750 µm^2^ FOV with 1200 A-scans/B-scan and 2400 B-scans/volume. At each lateral position, eight repeated B-scans (BM) were acquired using the shortest achievable interscan time (1.5 ms), resulting in a total acquisition time of 1.8 s per volume. To improve data reliability and minimize motion-related bias, at least five volumetric datasets were acquired at each imaging session (pre, post, 1 week, 1 month), and the best-quality volume was selected for subsequent analysis. Standard swept-source OCT reconstruction (k-space resampling, dispersion compensation, and Fourier transformation) was performed, followed by coherent buffer averaging [28]. to improve signal-to-noise ratio (yielding an effective 300 × 300 sampling density), motion correction, and OCTA computation from BM-scans. The details have been reported previously in Islam, Song, and Ju.[29].

## Results

Patient-level quantitative structural OCT parameters at four timepoints: Pre (− 1 hr), the post (+1 hour), week (7 ± 2), and the one-month follow-up (30 ± 7). Central retinal thickness (CRT), second band thickness (SBT), subfoveal choroidal thickness (SFCT), and subretinal fluid (SRF) height were recorded

A total of 5 patients (5 eyes) with cCSC were analyzed in the study. The mean age was 53.4 ± 11.4 years (range, 43 to 70 years), with 3 females. The mean duration of symptoms at the time of PDT was 23.4 ± 17.2 months (range, 3 to 48 months). Five patients were enrolled; however, analyzable PD-OCT data were available for 5 patients at baseline (pre-PDT), 5 patients immediately post-PDT, and 3 patients at the one-week and one-month follow-up. The mean best-corrected visual acuity (BCVA) in PDT-treated eyes was 0.385 ± 0.22 LogMAR. SRF was present in all treated eyes at baseline. The mean intraocular pressure was 11.6 ± 2.3 mmHg (OD) and 11.4 ± 1.9 mmHg (OS). Lens clarity was clear in 3 eyes and NS + 1 in 2 eyes (Table 1).

**Table 1:** Characteristics of the study participants and Eye dx = diagnosis dx

Patient ID	Age (years)	Sex	Eye PDT	Time since dx (month)	LogMAR OD	LogMAR OS	IOP OD	IOP OS	AC	Cornea	Lens status	Recurrent PDT	Subretinal Fluid	Spot Size (µm)
**1**	45	F	OS	24	0.176	0.216	12	11	d/q	Clear	Clear	N	Y	4000
**2**	48	F	OS	24	0.176	0.699	14	14	d/q	Clear	Clear	N	Y	4000
**3**	43	M	OD	18	0.497	0.020	13	10	d/q	Clear	Clear	N	Y	4000
**4**	61	M	OS	3	0.097	0.117	11	13	d/q	Clear	NS+1	N	Y	4000
**5**	70	F	OD	48	0.398	0.301	8	9	d/q	Clear	NS+1	Y	Y	5000

### Structural OCT parameters

Quantitative OCT analysis demonstrated consistent anatomic responses to PDT across patients (Table 2). SRF status was resolved or negligble in all cases at the 1-month visit as measured by structural B-scan, and therefore, a separate column for SRF height at that time point was not included in the table. At baseline, CRT ranged from 62 to 128 μm, SBT ranged from 16 to 129 μm, and subfoveal choroidal thickness (SFCT) ranged from 132 to 348 μm. SRF height was measurable in four eyes at baseline, ranging from 111 to 255 μm. Immediately after PDT, CRT values showed minimal fluctuation (86 to 130 μm), while SBT demonstrated early reductions (57 to 117 μm) except for smaller baseline values. SFCT remained relatively stable in the immediate post-treatment period (127 to 354 μm). SRF height measured immediately after PDT showed values comparable to baseline with only minor early fluctuations, consistent with the limited short-term anatomical changes observed in the other structural parameters.

**Table 2:** Quantitative structural OCT measurements

Patient ID	CRT Pre (μm)	CRT Post (μm)	CRT Week (μm)	CRT Month (μm)	SBT Pre (μm)	SBT Post (μm)	SBT Week (μm)	SBT Month (μm)	SFCT Pre (μm)	SFCT Post (μm)	SFCT Week (μm)	SFCT Month (μm)	SRF Height Pre (μm)	SRF Height Post (μm)	SRF Height Week (μm)
**1**	128	130	NA	NA	16	20	NA	NA	348	354	NA	NA	NA	NA	NA
**2**	86	94	90	94	129	117	105	22	275	286	255	240	255	238	123
**3**	87	86	86	90	68	71	43	19	305	355	337	338	142	146	51
**4**	62	84	NA	NA	125	100	NA	NA	184	181	NA	NA	111	91	NA
**5**	83	93	92	76	69	57	54	36	132	127	133	118	128	121	121

At the first weekly follow-up, available measurements showed continued decreases in SBT (43 to 105 μm) and SRF height (51 to 123 μm), with corresponding stabilization or mild increases in CRT. A one-month follow-up, there was decreased SFCT (118 to 240 μm) and reduced SBT (19 to 36 μm). SRF height remained lower than baseline in all eyes with available measurements (Table 2). Blinded Qualitative grading of choroidal hyperreflectivity by IB showed heterogeneous, non-directional changes between pre- and post-imaging [25, 30]. CC hyperreflectivity remained stable across time.[25, 30].

### PD-OCT

**Fig. 1:**
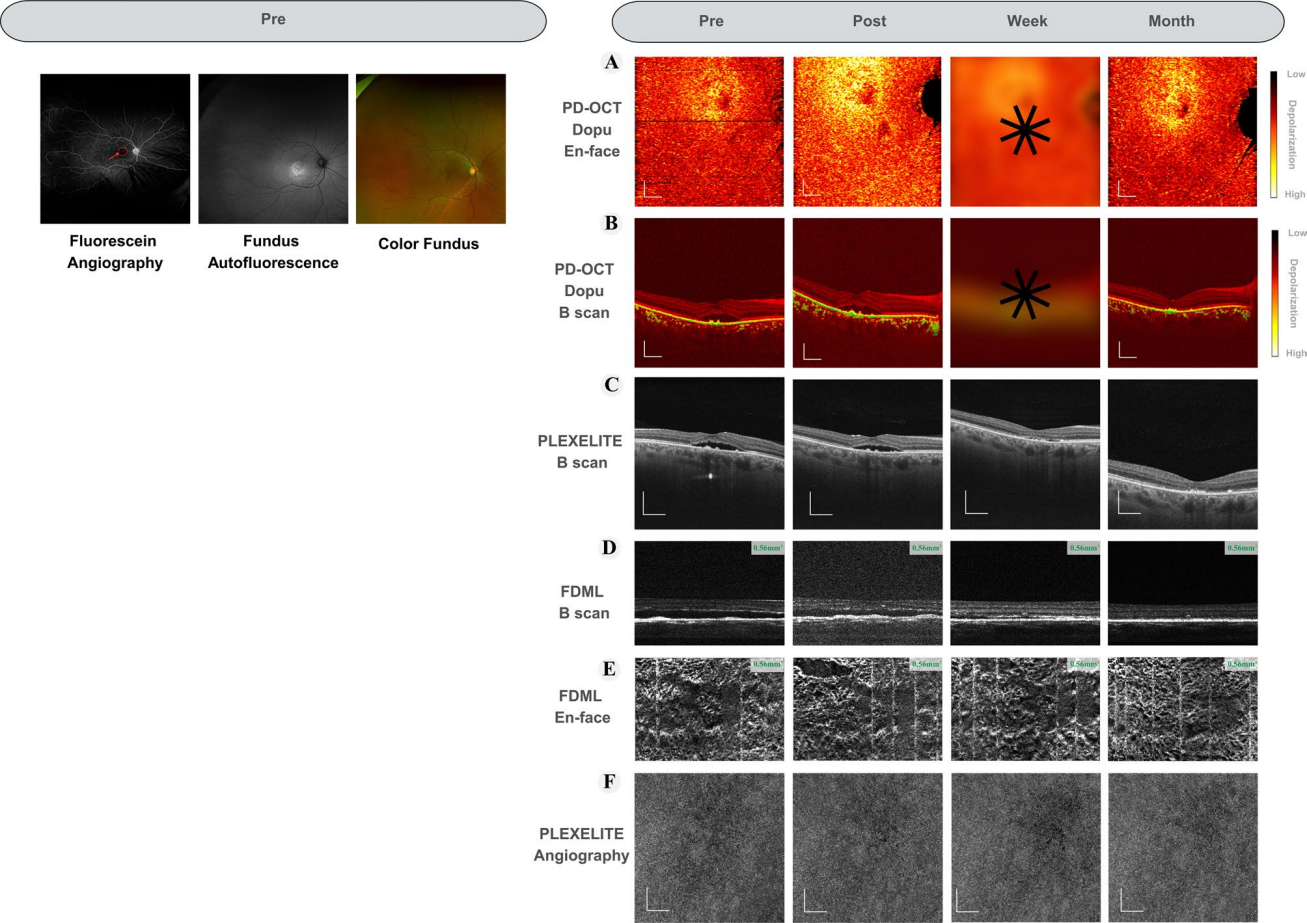
Multimodal imaging of a cCSC eye treated with PDT (scale bar = 1 mm). The left column shows baseline fluorescein angiography, fundus autofluorescence, and color fundus photography. The right grid shows (**A**) PD-OCT DOPU en-face maps (8x8mm) (**B**), PD-OCT DOPU B-scans (8x8mm) (**C**), PLEX Elite structural OCT B-scans (6x6mm) (**D**), FDML swept-source B-scans (750x750µm) (**E**), FDML en-face choriocapillaries images (750x750µm) (**F**), and PLEX Elite OCTA angiography(6x6mm) (**F**). Time points are pre, post, Week, and Month of PDT. * = no image available per protocol

Baseline fluorescein angiography, fundus autofluorescence, and color fundus images showed a well-defined area of leakage and corresponding subretinal fluid, highlighted by a red-circled region on angiography on the left panel (Fig. 1). Post-PDT, the DOPU en-face image revealed a reduction in DOPU signal within the 4,000-µm treatment zone compared to baseline (Fig. 1A). This is demonstrated by a shift toward the yellow signal. DOPU values re-approached the baseline by one month, with the signal shifting toward the red–dark portion of the scale, indicating increased DOPU (Fig. 1A). PD-OCT DOPU B-scans confirmed the same temporal change at the RPE–choroidal interface, showing reduced DOPU immediately after PDT and a return to the pre-treatment by 1 month; this pattern was consistent across all patients (Fig. S2). PLEX Elite structural OCT B-scans demonstrated the resolution of subretinal fluid over follow-up (Fig. 1C).

#### PD-OCT segmented analysis

**Fig. 2:**
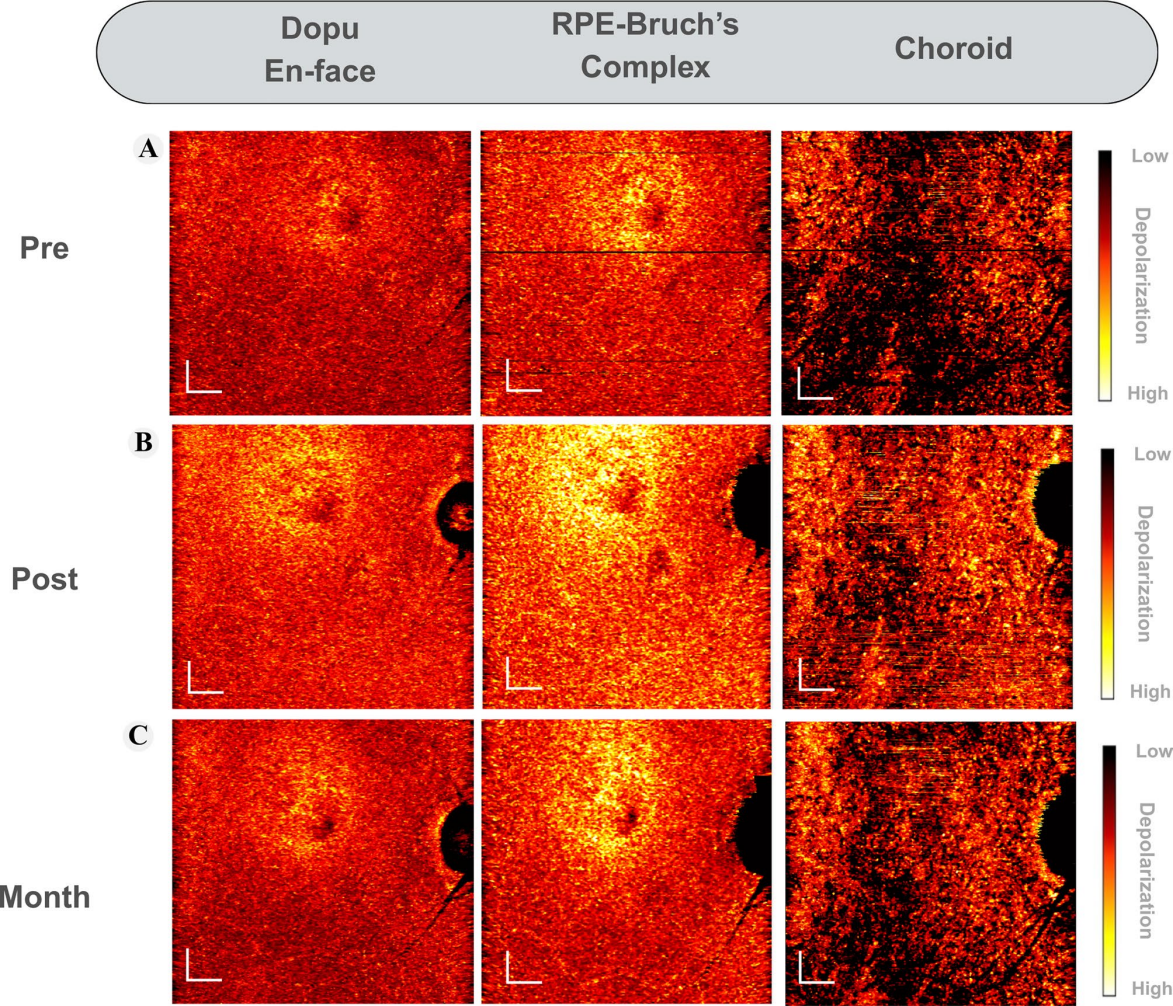
Segmented PD-OCT en-face depolarization maps at the DOPU, RPE–Bruch’s complex, and choroidal layers (scale bar = 1 mm). Panels show the (**A**) pre, (**B**) post, and (**C**) Month timepoints for three segmented layers: DOPU en-face RPE–Bruch’s complex and choroid

To localize the depolarization changes observed in Fig. 1, we performed manual segmentation of the volumes (Fig. 2). The RPE slab was segmented at the outer border (RPE–choriocapillaris boundary) to isolate melanin-rich structures at the RPE. Within this segmented zone (RPE-Bruch’s complex in Fig. 2A), there was a spatially confined decrease in DOPU signal within the PDT-treated macular region, mirroring the changes described above (Fig. 1A). In the subsequent Choroidal (RPE to Choroidoscleral junction) segmentation (Fig. 2), a similar but less confined pattern of decreasing DOPU is observed. This pattern remained consistent across all five cases, with a focal decrease in DOPU signal in the RPE–Bruch’s complex segmentation and a more diffuse, lower-intensity change in the choroidal segmentation (Fig. S2).

### FDML OCT

Using FDML OCT centered on the fovea, a 750 µm × 750 µm region was analyzed to assess choriocapillaris changes (Fig. 1E). Immediately after PDT, the scans showed an increase in dark, irregular flow patterns, and flow-deficient regions across the field (Fig. 1E). By one week, a distinct 300 µm × 150 µm flow void appeared in the superonasal quadrant, which persisted through the one-week follow-up (Fig. 1E). At 1 month, there was clear revascularization in the CC layer with the 300 µm × 150 µm flow void having resolved. These findings suggest a transient, localized reduction in choriocapillaris perfusion followed by partial revascularization over time.

## Discussion

Our study aimed to determine whether PDT produces measurable changes in depolarization signals in the human retina. CSC pathophysiology has been attributed to multiple mechanisms, including mechanical obstruction, inflammation, oxidative stress, autonomic dysfunction, mineralocorticoid receptor activation, and medication effects [4]. The prevailing hypothesis proposes that CC hyperpermeability and vascular congestion drive RPE barrier dysfunction, leading to subretinal fluid accumulation and visual loss [30, 31]. PDT mitigates this process by inducing choriocapillaris vascular remodeling [12]. PDT’s photochemical activation of verteporfin, generates reactive oxygen species, resulting in selective endothelial cytotoxicity and microthrombosis within abnormally permeable choroidal vessels [14, 32–34]. Within this framework, RPE melanin, composed predominantly of eumelanin, serves a protective role by absorbing light and quenching reactive oxygen species [35, 36]. We propose that PDT-induced oxidative activity alters melanin-associated depolarization, contributing to the decreased DOPU signal observed after treatment.

Our use of FDML in cCSC provides an additional non-invasive approach for visualizing the choriocapillaris and may serve as an adjunctive diagnostic imaging modality to other injection-based imaging [37]. FDML OCT overcomes depth-loss by combining a 1050-nm wavelength with shorter interscan phase-stable laser sweeps, resulting in greater penetration through the RPE, reduced sensitivity roll-off, and substantially improved signal-to-noise at the level of the choriocapillaris [29]. In our findings, we were able to visualize the choriocapillaris with fine detail and clearly demarcate the CC in cCSC. Although quantitative flow-deficit analysis or vascularity indices were not performed due to artefact post PDT, the images demonstrate a clear alteration in flow and choriocapillaris perfusion.

Based on a comprehensive literature review of all relevant databases conducted on Feb 4, 2026, this is the first clinical study to evaluate depolarization changes using PD-OCT or FDML before and after photodynamic therapy in patients with cCSC.

### Interpretation of findings

Our observations suggest that PDT-induced oxidative stress may influence melanin-associated depolarization signals in PDT-treated areas. However, the precise mechanism underlying the observed decrease in DOPU remains uncertain. Based on available experimental and histopathologic literature, several biologically plausible processes may contribute to these changes [25, 38–41]. Our results indicated that the most prominent DOPU signal alterations were observed at the RPE–Bruch’s membrane complex, suggesting that mechanisms may operate within a shared framework in which PDT-induced oxidative activity influences RPE melanin through processes such as melanin structural alteration and migration of pigment-laden macrophages.[25, 38–41].

On the molecular level, melanin’s structure depolarizes light and lowers DOPU value [39]. The depolarization arises from the indole- and tyrosine-derived chromophore structures within melanin [39, 40]. Notably, PDT’s induction of singlet oxygen may lead to the oxidation of double bonds in tyrosine- and indole-derived rings in melanin’s structure [40]. This oxidation may cleave indole rings to form smaller pyrrolic structures, potentially altering melanin’s interaction with polarized light and the observed DOPU signal [39, 40]. On a structural level, PDT is known to damage melanosomes when used as targeted therapy; however, half-dose PDT does not alter the structure of melanosomes relative to untreated control areas in histopathological studies [41]. One could postulate that the modification of the chemical structure of melanin without substantially altering the overall melanin content leads to a lower DOPU signal.[39–41].

Another mechanism that may contribute is CSC-induced choroidal congestion and possible macrophage migration into the choroid following congestion induction [1]. The choroid exhibits variable DOPU signals depending on the subject, which may in part be related to migration of pigmented cells and phagocytosis of melanin granules [35]. These pigment-loaded macrophages are a potential source of light depolarization [39]. Notably, PDT has been shown to cause a transient macrophage suppression followed by an increase in density [41]. Histopathologic studies of PDT-treated choroidal neovascular tissue have demonstrated a time-dependent inflammatory response characterized by early suppression followed by increased macrophage infiltration, suggesting that immune-cell recruitment may occur during post-treatment remodelling [42]. Within this framework, macrophages capable of phagocytosing melanin granules could transiently redistribute pigment within the RPE–choriocapillaris complex, potentially modifying the spatial arrangement of depolarizing structures detected by PD-OCT [42]. However, direct evidence linking macrophage-related processes to the DOPU changes observed in CSC is currently lacking, and this mechanism should therefore be considered speculative.

The hyperreflective patterns observed before and after PDT were analysed as a potential confounding source of polarization. The heterogeneity and lack of consistent increase or decrease in hyperreflectivity indicate that PD-OCT DOPU changes are less likely to be driven by gross choriocapillaris reflectivity alterations.[25].

Several limitations applicable to this study warrant consideration. First, the sample size in the current study was small, which limits generalizability; however, all patients demonstrated a layer-specific decrease in DOPU that was temporally related to PDT treatment and biologically plausible given singlet oxygen’s effects on melanin. Furthermore, two patients did not complete follow-up imaging, resulting in a reduced number of analysable cases at later time points. In addition, the global verteporfin shortage during the study period further constrained patient recruitment. Another limitation of the present study is the absence of quantitative DOPU measurements using PD-OCT, which precluded calculating an empirical effect size and conducting a formal power analysis based on the observed data. Moreover, FDML imaging requires relatively stable fixation, and patients after PDT often had greater difficulty maintaining fixation, which resulted in motion-related artifacts in some images. To address this limitation, future studies will incorporate multi-volume acquisition followed by 3D feature-based registration and volume averaging to reduce motion artifacts and improve image quality. Lastly, given the exploratory nature of this study and the absence of normative DOPU reference values, the clinical utility of DOPU as a biomarker remains to be established.

## Conclusion

Half-dose PDT in chronic CSC induced measurable DOPU signal decrease in RPE melanin detectable by PD-OCT. The decrease in DOPU signal was most pronounced in the 4,000 µm laser-treated region centered at the fovea, consistent with selective choriocapillaris occlusion and vascular remodeling. These findings are consistent with localized oxidative modification of indolic chromophores in melanin. DOPU signal is measured in PD-OCT and can be a non-invasive biomarker for RPE melanin integrity, with potential applications in evaluating PDT-induced changes in other retinal pathologies.

## Electronic supplementary material

Below is the link to the electronic supplementary material.

Supplementary Material 1

Supplementary Material 2

Supplementary Material 3

## Data Availability

No datasets were generated or analysed during the current study.

## Declarations

### AI use statement

During the preparation of this work, the author(s) used ChatGPT (OpenAI) to improve clarity, organization, and efficiency in drafting tables and summaries. After using this tool/service, the author(s) reviewed and edited the content as needed and take(s) full responsibility for the content of the published article.

## Abbreviations

cCSC: Chronic central serous chorioretinopathy
ROS: Reactive oxygen species
FDML: Fourier-domain mode-locked optical coherence tomography
PD-OCT: Polarization-diversity optical coherence tomography
PS-OCT: Polarization-sensitive optical coherence tomography
FA: Fluorescein angiography
DOPU: Degree of polarization uniformity
RPE: Retinal pigment epithelium
PDT: Photodynamic therapy
