# Supplementary figures and images for: Longitudinal polarization diversity optical coherence tomography before and after photodynamic therapy in chronic central serous chorioretinopathy: a prospective clinical study
Source: Int J Retina Vitreous. 2026 May 11;12:95. doi: 10.1186/s40942-026-00857-8 (PMC13335328; doi:10.1186/s40942-026-00857-8)

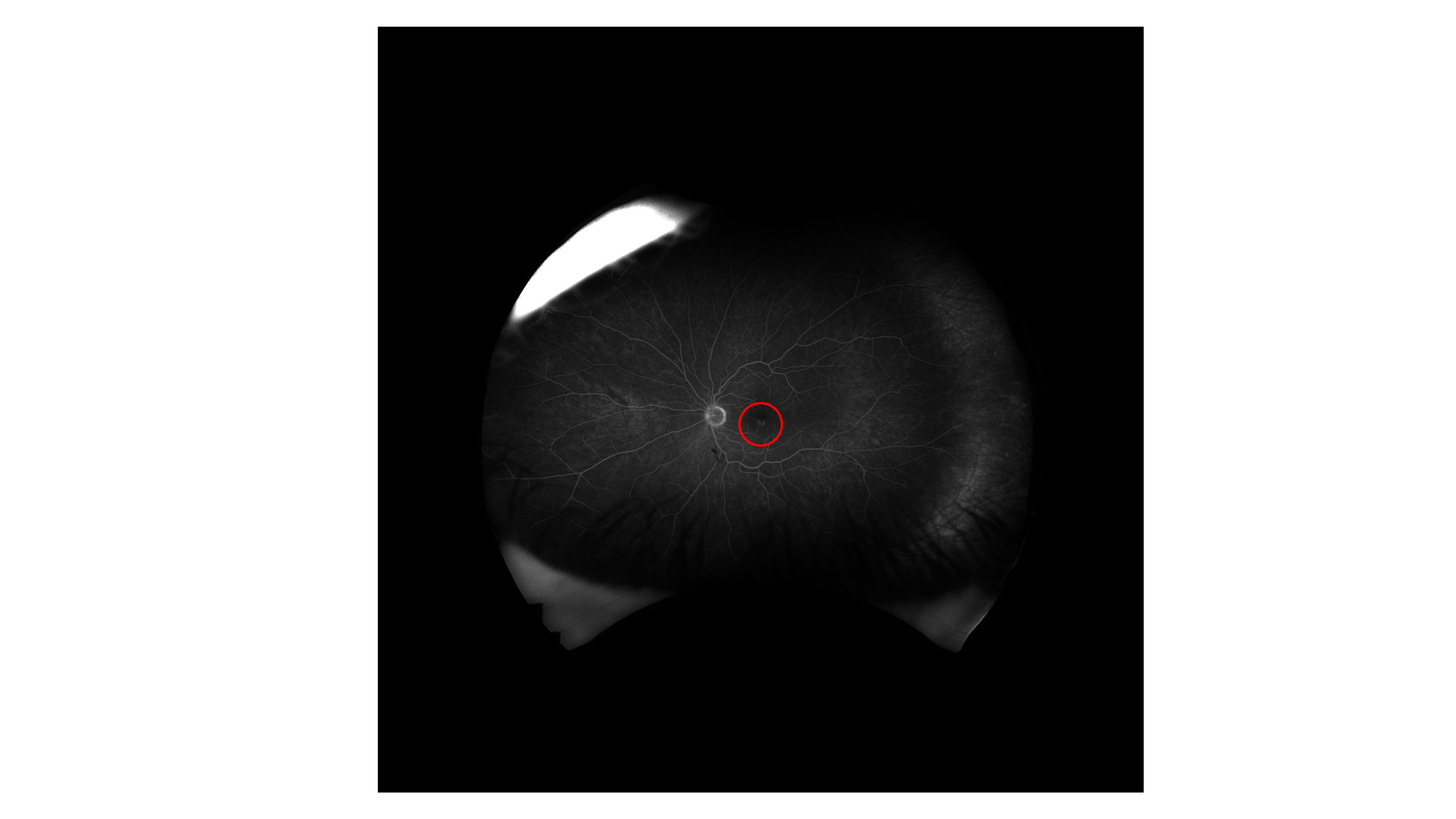

Supplement: Supplementary file 1 — Supplementary Material 1 [file 40942_2026_857_MOESM1_ESM.png]

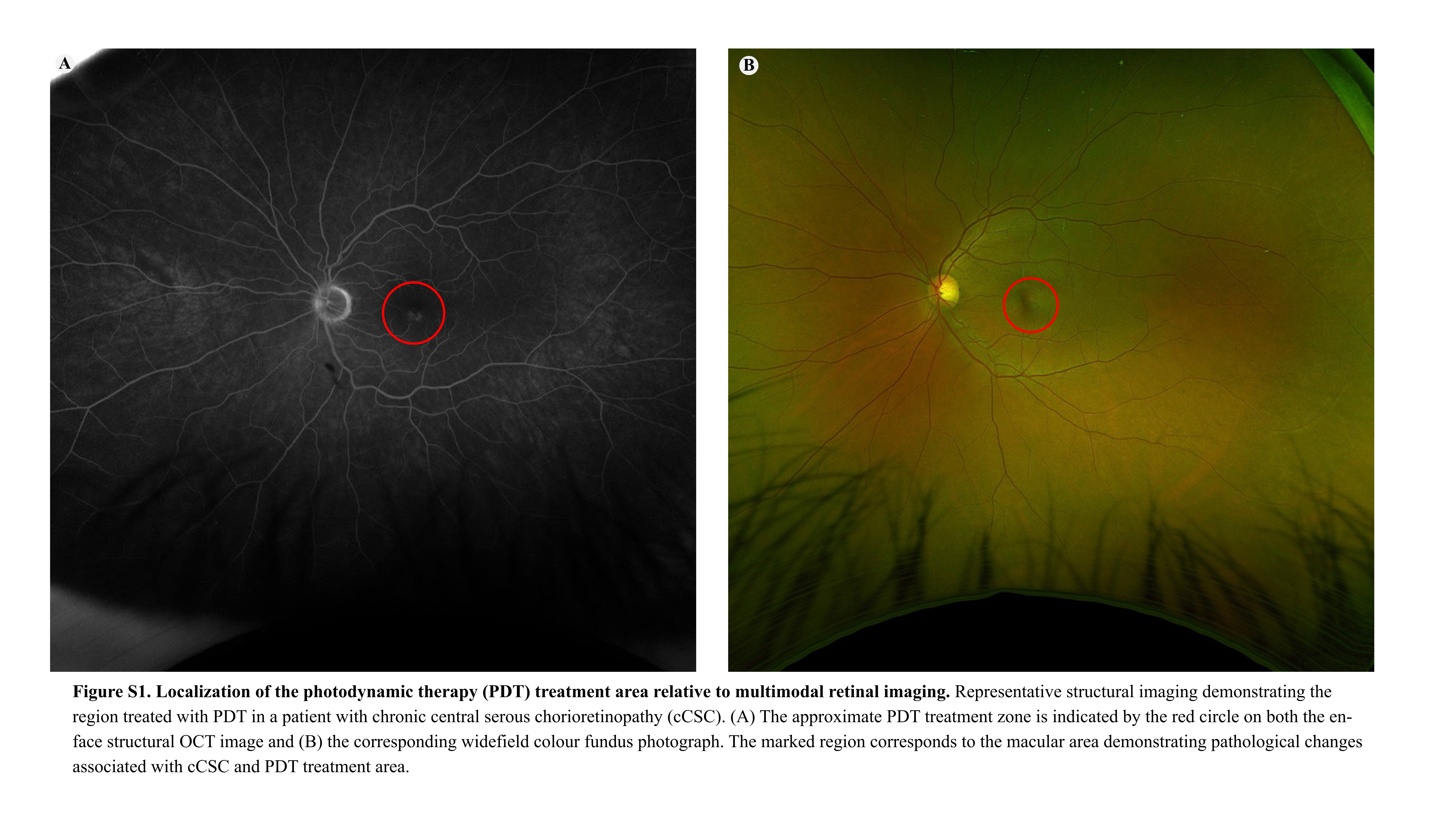

Supplement: Supplementary file 2 — Supplementary Material 2 [file 40942_2026_857_MOESM2_ESM.png]

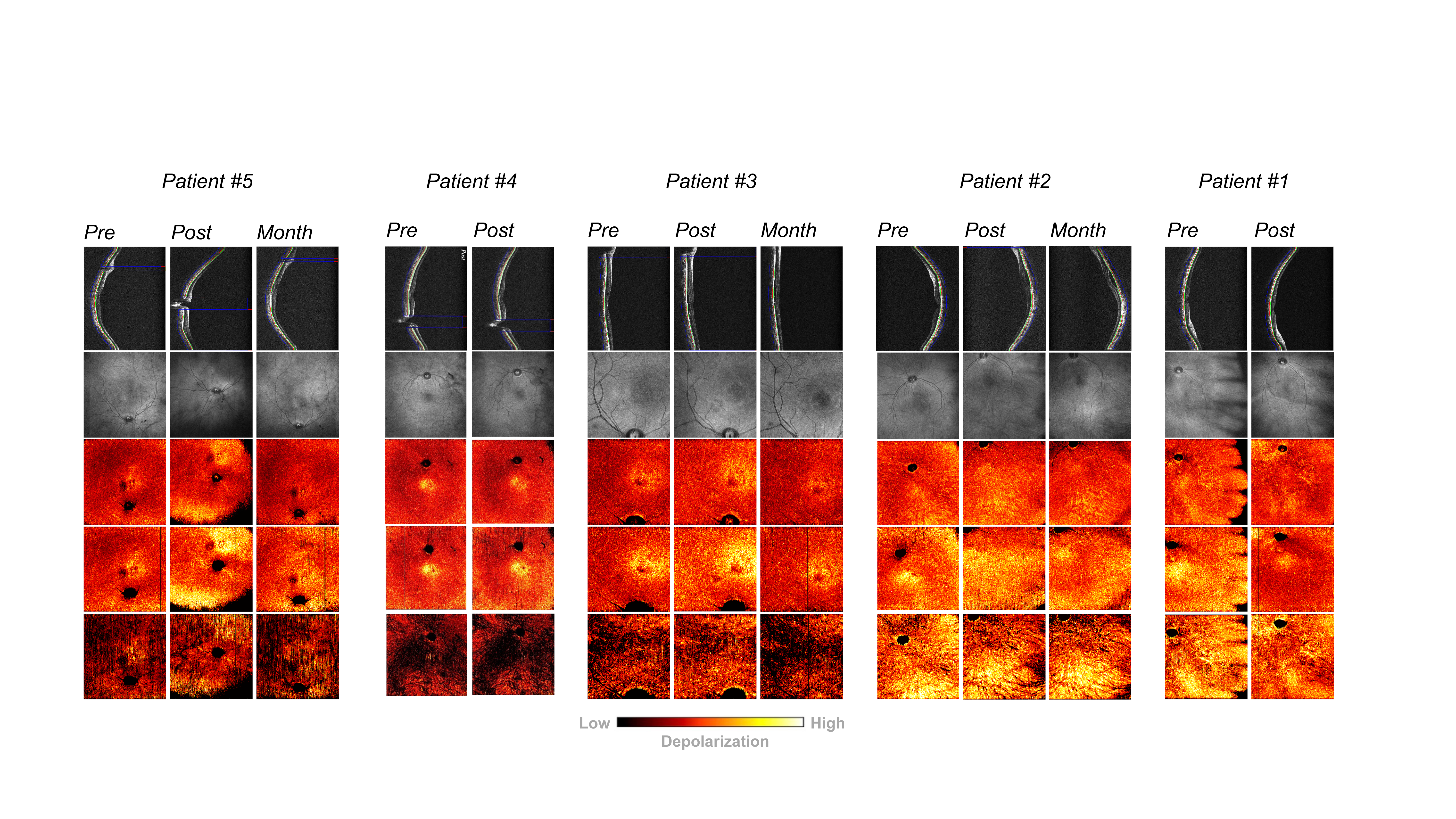

Supplement: Supplementary file 3 — Supplementary Material 3 [file 40942_2026_857_MOESM3_ESM.png]
